# Supplementary material for: Environmental regulation and corporate tax avoidance—Evidence from China
Source: PLoS One. 2022 Jan 13;17(1):e0261037. doi: 10.1371/journal.pone.0261037 (PMC8757985; doi:10.1371/journal.pone.0261037)
Supplement: S1 Appendix — (DOCX) [file pone.0261037.s001.docx]

Appendix A

**Table A1. Variable definitions.**

| **Variable** | **Definitions** |
| --- | --- |
| $TA1$ | Tax rate difference, a direct measure of corporate tax evasion, the difference between (income tax expenses/ pre-tax accounting profit) and the nominal rate. |
| $TA2$ | Tax rate difference, a direct measure of corporate tax evasion, the difference between (income tax expense - deferred income tax expense)/(pre-tax accounting profit - deferred income tax expense/nominal rate) and the nominal rate . |
| Treat | A dummy variable that is equivalent to 1 if the registered place of one company is in Beijing, Tianjin, Shanghai, Chongqing, Hubei, and Guangdong province, and zero otherwise. |
| $\mathrm{Post}$ | A dummy variable that is equivalent to 1 if the year ≥ 2013, and zero otherwise. |
| $\mathrm{Lev}$ | Firm leverage, the ratio of total debt to total assets. |
| $\mathrm{Size}$ | Firm size, the logarithm of total assets. |
| $\mathrm{NetFi}$ | Fixed assets on assets, the ratio of fixed assets to total assets. |
| $\mathrm{NetIn}$ | Intangible assets on assets, the ratio of intangible assets to total assets. |
| $\mathrm{Roa}$ | Return on assets, the ratio of net profit to total assets. |
| $\mathrm{Age}$ | Firm age, one plus the current year and minus the establishing year. |
| $\mathrm{Foac}$ | A dummy variable that equals to one if the company employs the four largest accounting firms and zero otherwise |
| $\mathrm{Soe}$ | A dummy variable that is equivalent to 1 if it is a state company and zero otherwise |

**Table 2A. Robustness and endogenous test.**

| **Panel A** | **TA2** | | | |
| --- | --- | --- | --- | --- |
|  | **(1)** | **(2)** | **(3)** | **(4)** |
| $\mathrm{Treatpost}$ | -0.0140*** | -0.0088* | -0.0104* | -0.0116** |
|  | (-2.5912) | (-1.8022) | (-1.9004) | (-2.4309) |
| Exc_Tax_Pol | YES |  |  |  |
| Con_Tim_Trend |  | YES |  |  |
| Exc_Eig_Reg |  |  | YES |  |
| Con_SO2_Pol |  |  |  | YES |
| $\mathrm{Obs}$ | 7577 | 9390 | 6952 | 9390 |
| $Adj\_R2$ | 0.429 | 0.417 | 0.441 | 0.414 |
| **Panel B** | **TA2** | | |  |
|  | **(5)** | **(6)** | **(7)** |  |
| $\mathrm{Treat}*\mathrm{Post}$ | -0.0141*** | -0.0214*** | -0.0124** |  |
|  | (-2.7045) | (-3.2264) | (-2.2711) |  |
| Sam_2011_2014 | YES |  |  |  |
| PSM-DID |  | YES |  |  |
| Two_DID |  |  | YES |  |
| Cont_Vars | YES | YES | YES |  |
| Firm/ Year FE | YES | YES | YES |  |
| $\mathrm{Obs}$ | 4910 | 3967 | 2982 |  |
| $Adj\_R2$ | 0.519 | 0.419 | 0.426 |  |

^a^ T-statistics of clustering to enterprises are shown in parentheses

^b^ ***, **, and * indicate significance at the 1%, 5%, and 10% levels, respectively.

**Table 3A. Heterogeneity analysis.**

|  |  | | **TA2** | | |  | |
| --- | --- | --- | --- | --- | --- | --- | --- |
|  | **Ownership structure** | | **Firm size** | | | **Industry competition** | |
|  | **NSOE** | **SOE** | **Small** | | **Big** | **High** | **Low** |
|  | **(1)** | **(2)** | **(3)** | | **(4)** | **(5)** | **(6)** |
| $\mathrm{Treatpost}$ | -0.0185*** | -0.0104 | -0.0012 | -0.0229*** | | -0.0120* | -0.0119* |
|  | (-2.6180) | (-1.5995) | (-0.2058) | | (-3.0170) | (-1.7122) | (-1.7527) |
| Cont_Vars | YES | YES | YES | | YES | YES | YES |
| Firm/ Year FE | YES | YES | YES | | YES | YES | YES |
| $\mathrm{Obs}$ | 4283 | 5049 | 4592 | | 4598 | 4433 | 4957 |
| $Adj\_R2$ | 0.412 | 0.440 | 0.435 | | 0.448 | 0.409 | 0.419 |

^a^ T-statistics of clustering to enterprises are shown in parentheses

^b^ ***, **, and * indicate significance at the 1%, 5%, and 10% levels, respectively.
